# A real-time phase-space beam emittance monitoring system

**DOI:** 10.1107/S1600577519005423

**Published:** 2019-06-18

**Authors:** Nazanin Samadi, Xianbo Shi, Les Dallin, Dean Chapman

**Affiliations:** a Physics and Engineering Physics, University of Saskatchewan, 116 Science Place, Saskatoon, SK, Canada S7N5E2; b Advanced Photon Source, Argonne National Laboratory, 9700 South Cass Avenue, Lemont, IL 60439, USA; c Canadian Light Source, 44 Innovation Boulevard, Saskatoon, SK, Canada S7N2V3

**Keywords:** beam emittance monitor, X-ray diffraction, phase space, diagnostics and feedback

## Abstract

An electron beam position and angle monitoring system, ps-BPM, has been shown to be able to measure the electron source position and angle at a single location in a beamline at a synchrotron source. This system uses a mono­chromator to prepare a photon beam whose energy is at that of the *K*-edge of an absorber filter. The divergence of the beam from the source gives an energy range that will encompass the *K*-edge of the filter. A measurement of the centre of the monochromatic beam and the *K*-edge location through the absorber filter gives the position and angle of the electron source. Here, it is shown that this system is also capable of measuring the source size and divergence at the same time. This capability is validated by measurement as the beam size in the storage ring was changed and by ray-tracing simulations. The system operates by measuring the photon beam spatial distribution as well as a *K*-edge filtered beam distribution. These additional measurements result in the ability to also determine the electron source size and divergence.

## Abstract 2

An electron beam position and angle monitoring system, ps-BPM, has been shown to be able to measure the electron source position and angle at a single location in a beamline at a synchrotron source. This system is now shown to be capable of measuring the source size and divergence simultaneously.

## 1. Introduction   

The emittance of the electron beam is a fundamental property of synchrotron sources as it describes the area of the transverse phase space of the electron source (product of the size and divergence) (Buon, 1992 ▸). Measuring the electron source size and divergence is important and becoming ever more critical as the next generation of light sources are being planned and built (Eriksson *et al.*, 2014 ▸; Tavares *et al.*, 2014 ▸), such as implementing a multi-bend achromat (MBA) lattice (Einfeld *et al.*, 2014 ▸). These new machines are focusing on achieving the smallest possible emittance ideally below the emittance of the photon radiation to deliver a diffraction-limited beam. For example, at the Advanced Photon Source (APS) the upgrade source emittance will be reduced from 3.1 nm rad to 42 pm rad (Borland *et al.*, 2018 ▸). The source size and divergence of these new machines are typically in the range of a few micrometres and microradians. The increased stability requirement for these new sources demands real-time monitoring of the source position, angle, size and divergence to characterize and correct the source. There are different ways of measuring source size, which can be categorized as direct imaging and diffraction-based techniques.

Direct imaging systems include pinhole measurements (Elleaume *et al.*, 1995 ▸; Thomas *et al.*, 2010 ▸) and techniques using focusing optics like compound refractive lenses (Weitkamp *et al.*, 2000 ▸), Fresnel zone plates (Iida *et al.*, 2003 ▸; Takano *et al.*, 2006 ▸) and Kirkpatrick–Baez mirrors (Renner *et al.*, 1996 ▸).

The traditional pinhole measurement is the most common technique used at the existing third-generation synchrotron facilities to give real-time information on the electron beam size in both transverse directions. The pinhole technique is simple and requires no focusing optics. However, it has a limited resolution (∼10 µm), which makes it impractical for new high-brightness sources.

The diffraction-based methods utilize the spatial coherence of the source. To be able to analyze the contrast of the interferogram for these measurements requires high intensity compared with direct imaging systems. Available methods primarily rely on interferometry, such as double-slit (Mitsuhashi, 1999 ▸; Naito & Mitsuhashi, 2006 ▸; Corbett *et al.*, 2017 ▸), grating (Guigay *et al.*, 2004 ▸; Shi *et al.*, 2014 ▸) and multi-lens X-ray interferometry (Lyubomirskiy *et al.*, 2016 ▸; Kohn *et al.*, 2000 ▸). Another recent interferometry method is based on π polarization measurements (Andersson *et al.*, 2008 ▸).

Most of the existing ways of measuring the emittance are only capable of measuring the size of the electron source with no information about the divergence except for a pinhole array system (Peatman & Holldack, 1998 ▸) which is limited in resolution.

The ps-BPM system was introduced previously (Samadi *et al.*, 2015 ▸), where the electron beam angle and position were measured simultaneously at a single location in a synchrotron beamline. In this paper, we will describe how that system can also be used to extract information about the electron beam source size and divergence and thus the potential for a powerful real-time diagnostic tool.

## 2. ps-BPM system   

**Figure 1:**
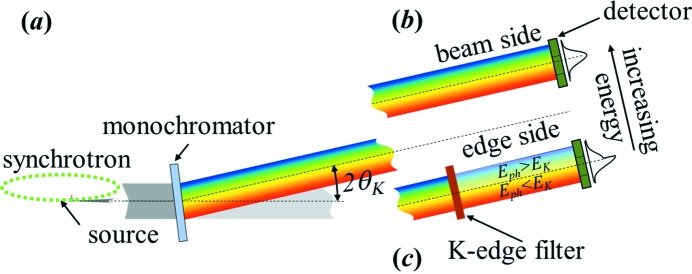
Schematic of the ps-BPM system. (*a*) Side view of the source and monochromator (single Laue). Horizontally separated (*b*) unfiltered beam side and (*c*) filtered edge side of the photon beam on the same detector. The example intensity profile on the right side of the figure in (*b*) shows the unfiltered photon distribution, and in (*c*) shows the intensity change in the vertical plane after the filter with the *K*-edge energy in the middle of the beam.

A schematic view of the ps-BPM system is shown in Fig. 1 ▸. Fig. 1(*a*) ▸ shows the source and monochromator. The monochromator shown is a single-crystal transmission type or Laue monochromator. The work shown here is primarily from a double-crystal reflection or Bragg-type monochromator (DCM) (Golovchenko *et al.*, 1981 ▸). It has been shown that both types of monochromators can be used; however, the Laue-type monochromator is more tolerant to heat loading from the source though typically limited to higher X-ray energies (≳20 keV).

Figs. 1(*b*) and 1(*c*) ▸ show two horizontally separated portions of the beam from the monochromator. Fig. 1(*b*) ▸ shows the unfiltered beam (beam side) plus the detector and Fig. 1(*c*) ▸ shows the *K*-edge filter (edge side) on the same, but horizontally displaced, part of the detector. The system records the photon beam profile produced by a bending-magnet (BM) source monochromated by a crystal. The photon energy is tuned to the absorption *K*-edge of a selected filter, which partially covers the horizontal BM fan. The filtered and unfiltered beam profiles are recorded by an area detector and are fitted to appropriate functions. The vertical electron beam position and angle information are extracted from the parameters of these fits (Samadi *et al.*, 2015 ▸).

In addition, the vertical electron beam source size and divergence (emittance) information is carried in the measured widths of the unfiltered and filtered beam. These measured widths include other contributions that will now be discussed.

### 2.1. Contributions to the unfiltered beam width   

The measured spatial photon beam width on the unfiltered beam side, 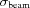, on the detector at a distance *D* from the source includes a convolution of the vertical electron source size, 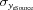, the divergence of the electron source, 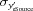, and the opening angle of the photon beam (Schwinger, 1949 ▸), 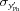. Here 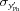 is modelled as a Gaussian distribution which is a good approximation when the photon energy is well above the critical energy of the source. This relation is shown in equation (1) where the contributions to the beam width are all assumed to be Gaussian distributions, so they add in quadrature,
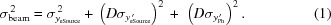
The divergence of the electron beam at the source can be calculated as
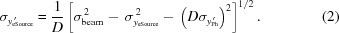
From equation (2), measurements of 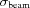, 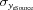 and 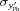 are required in addition to *D* to determine 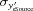.

**Figure 2:**
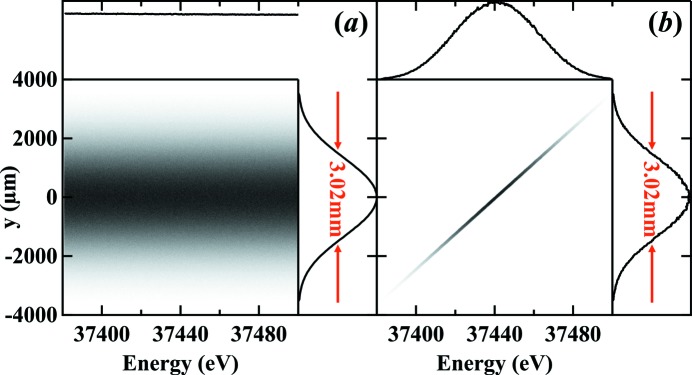
Simulated vertical beam profile on the detector at 26 m from the source as a function of photon energy (*a*) without and (*b*) with the monochromator. The FWHM size of the two profiles shows that the presence of the monochromator has no effect on the detected beam size.

Equation (1) has no contributions from the monochromator which can be understood by considering the DuMond diagram (DuMond, 1937 ▸) for a monochromator with a synchrotron source. This is demonstrated in Fig. 2 ▸ which was modelled with a ray-tracing simulation around the *K*-edge of barium at 37.441 keV using *ShadowOui* (Rebuffi & Sanchez del Rio, 2016 ▸) in the OASYS environment (Rebuffi & Sanchez del Rio, 2017 ▸).

The determination of 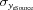 arises from the *K*-edge measurement and is described next.

### 2.2. Contributions to the measured *K*-edge width of the filtered beam   

The filtered beam profile measured on the detector has an energy distribution because of the vertical divergence of the beam which allows the effects of the *K*-edge to be observed spatially. For this discussion the focus will be on the vertical distribution of this measured edge.

The *K*-edge in the measurements appears as a smoothed step function and is modelled as an error function (an integral of a Gaussian). A derivative of the data results in a Gaussian-type peak.

The measured *K*-edge width of this peak, 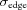, includes the vertical electron source size, 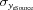, the natural angular width of the *K*-edge, 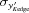, and the intrinsic angular width of the monochromator (Warren, 1990 ▸; Zachariasen, 1945 ▸), 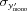, taken in quadrature; this is shown in equation (3), 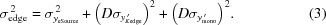
From equation (3),
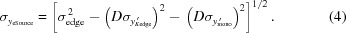



#### 2.2.1. Natural angular width of the *K*-edge and the monochromator   

**Figure 3:**
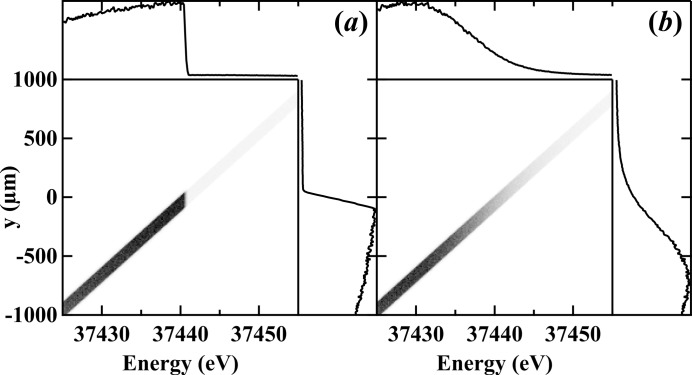
Simulated vertical beam profile as a function of photon energy after (*a*) a filter with sharp absorption edge at 37.441 keV and (*b*) a barium filter with a 13.2 eV *K*-edge width. The edge size in (*a*) shows the effect of the monochromator broadening, and in (*b*) shows the broadening effect of both the monochromator and the filter *K*-edge.

The *K*-edge is a characteristic absorption feature of an element where the absorption changes for photon energies at and above the binding energy, 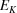, of inner-shell electrons. The absorption around this edge is broadened by the core-hole lifetime. The core level broadening can be assumed as a Gaussian function with a width of 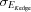 in energy (Babanov *et al.*, 1998 ▸). Because of the energy dispersion effect of the monochromator crystal, the energy edge width can be converted to an angular width given by
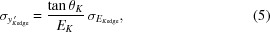
where 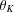 is the Bragg angle of the crystal at 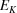. Fig. 3 ▸ shows the calculated photon beam profile with a barium filter whose *K*-edge is 37.441 keV with a Si (2,2,0) DCM. The ray-tracing calculations were made at a distance *D* from the source with a filter having [Fig. 3(*a*) ▸] both a sharp width (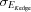 = 0 eV) *K*-edge and [Fig. 3(*b*) ▸] a finite width (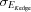 = 5.6 eV). The *K*-edge broadening of the energy scale is projected onto the spatial scale, 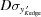, on the detector based on equation (5). One should note that for the filter with a sharp *K*-edge [Fig. 3(*a*) ▸] the spatial edge width on the detector is non-zero due to the contribution of the monochromator crystal, 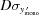.

#### 2.2.2. Electron source size effects   

**Figure 4:**
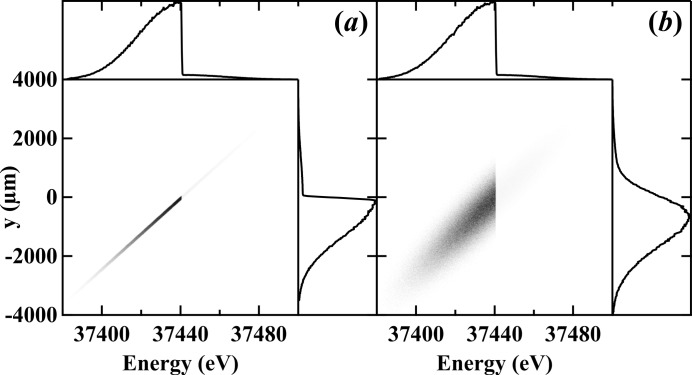
Simulated vertical beam profile as a function of photon energy to show the effect of the electron source size on the filtered edge side of the beam. (*a*) An electron source with zero emittance and (*b*) a Gaussian electron source with 510 µm vertical size.

As was shown in the 2015 publication where the beam position and angle were determined by the ps-BPM system, the edge location identifies with the position of the electron source (Samadi *et al.*, 2015 ▸). For this reason, the spatial broadening of the *K*-edge is a direct measurement of the source size. Fig. 4 ▸ illustrates this effect for the case where 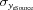 = 0 [Fig. 4(*a*) ▸] and 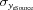 = 500 µm [Fig. 4(*b*) ▸]. Note the broadening of the *K*-edge as projected on the *y*-axis.

The results of Section 2 show that by having the measurements of the *K*-edge filtered side and unfiltered beam side available at the same time one can compute the vertical size and divergence of the electron beam if the contributions from the terms 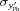, 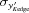 and 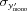 along with the source-to-detector distance, *D*, are known.

## 3. Measurements and results   

### 3.1. Experimental setup   

Experiments were carried out at the Biomedical Imaging and Therapy bend magnet (BMIT-BM) beamline (Wysokinski *et al.*, 2007 ▸, 2013 ▸) at the Canadian Light Source (CLS) with photon beam energy at the barium (37.441 keV) *K*-edge selected using a silicon (2,2,0) DCM. A combination of 0.1 mm aluminium and 0.25 mm copper filters was used to reduce the heat load on the monochromator to less than 2 W over the full width of the beam and normal storage ring operating conditions.

Part of the horizontal width of the beam was filtered with a 60 mg cm^−2^ barium solution absorber. Both filtered and unfiltered parts of the beam were intercepted by a flat-panel Hamamatsu (Hamamatsu Photonics, Hamamatsu City, Shizuoka Prefecture, Japan) detector with a pixel size of 100 µm × 100 µm and 33 Hz frame rate to record the images. For this application the detector was 26 m from the source.

### 3.2. Data analysis   

The unfiltered beam side and filtered edge side of the beam were recorded simultaneously and analyzed using programs written in IDL (Interactive Data Language; ITT Visual Information Solutions, Boulder, CO, USA). The detector dark response (image of the detector with no beam) was subtracted from both the unfiltered and *K*-edge filtered sides of the data. The data were then averaged over a select horizontal region to generate one-dimensional vertical beam profiles (unfiltered and *K*-edge filtered). The quantity 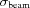 in equation (2) was obtained by fitting the profile from the unfiltered beam side to a Gaussian.

The *K*-edge filtered profile was then normalized by the unfiltered profile. The derivative of the negative logarithm of this normalized profile was also fit to a Gaussian. The width of the fit (RMS value) is the factor 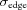 in equation (3). The contributions of the natural angular width of the *K*-edge and the monochromator were determined through a numerical error minimization procedure as described in the *Results* [Sec sec3.3] section. This total contribution was then subtracted in quadrature from 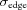 based on equation (4) to solve for the vertical electron beam size at the source, 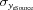.

The divergence of the photon beam was calculated and fit to a Gaussian to solve for 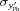. Referring to equation (2), the divergence of the electron beam at the source, 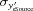, was then calculated by subtracting in quadrature 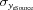, which was obtained from the *K*-edge filtered side and the calculated 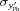.

**Figure 5:**
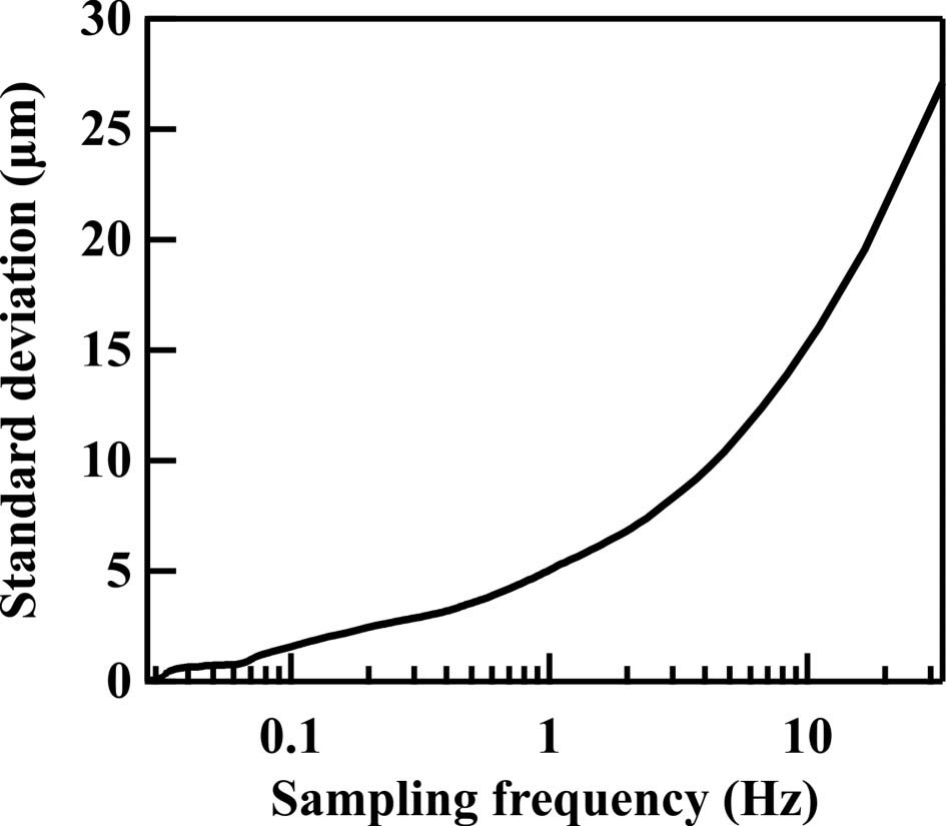
RMS error of the extracted electron source size as a function of sampling frequency.

The RMS fitting error was found by averaging the data over various time scales to simulate different sampling frequencies. The RMS error values are plotted as a function of sampling frequency in Fig. 5 ▸. To achieve higher accuracy, longer integration time (lower sampling frequency) is needed. A sampling frequency of 0.33 Hz was chosen which gave an RMS value of less than 5% of the electron beam size across all measurements. Note that this sampling frequency was limited by the achievable intensity across the small photon beam width that was taken.

### 3.3. Results   

Measurements were performed during special operation shifts at the CLS while the size of the electron beam was changed by altering the vertical–horizontal coupling using different currents in skew quads. This method of changing the source size and divergence was chosen since it does not change the machine beta-functions in the storage ring.

**Figure 6:**
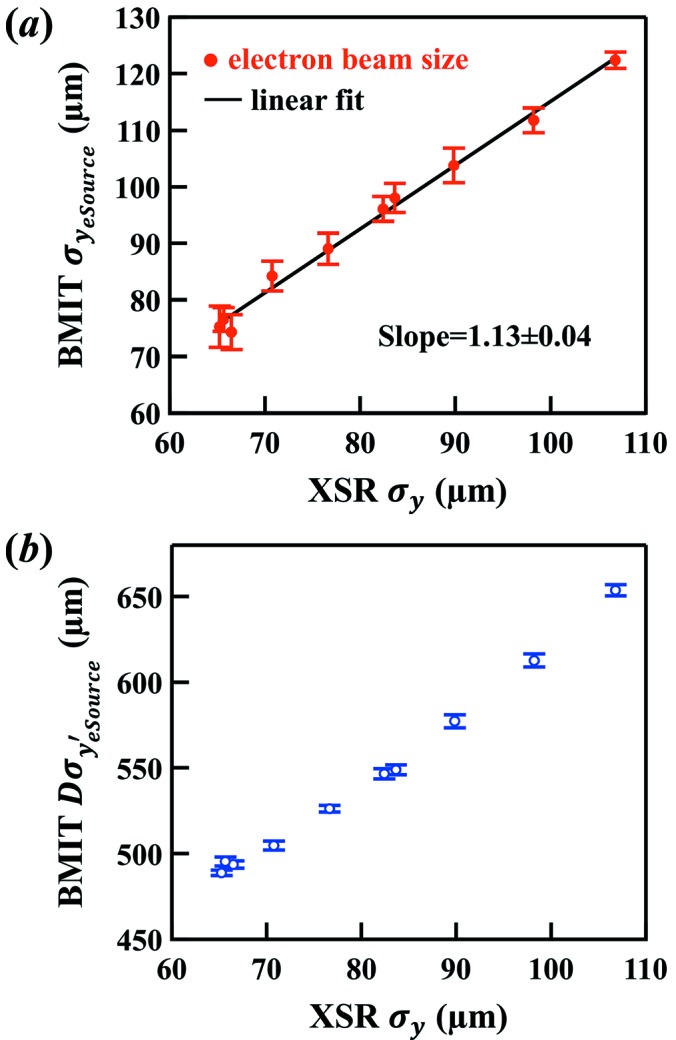
Measured (*a*) electron source size and (*b*) electron source divergence using the ps-BPM system at the BMIT beamline. The horizontal axis is the size measured using a pinhole setup at the XSR diagnostic beamline.

The data were compared with measurements recorded with a 30 µm pinhole camera at a diagnostic BM beamline, X-ray Synchrotron Radiation (XSR) (Bergstrom & Vogt, 2008 ▸). The pinhole measurements were made at the same time as the ps-BPM measurements. The measured electron source size with the ps-BPM system at the barium *K*-edge is shown in Fig. 6(*a*) ▸ compared against the source size measured by the pinhole. Since the pinhole cannot measure the divergence of the source, the measured divergence with the ps-BPM system is also plotted against the size values from the pinhole measurement [see Fig. 6(*b*) ▸]. Each data point was obtained with a sampling rate of 0.33 Hz over a total period of 40 s.

The size contribution by the natural angular width of the *K*-edge, 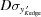, and the monochromator, 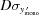, corresponds to a vertical width of 386.6 µm on the detector when added in quadrature. This vertical width was determined by a numerical minimization procedure involving a fit to measured ps-BPM data as a function of XSR values to achieve the best linear zero-intercept result while varying the vertical width parameter.

The monochromator contribution, 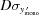, can be calculated using the intrinsic angular width for the monochromator crystals (Warren, 1990 ▸; Zachariasen, 1945 ▸) which is equal to 59.4 µm on the detector (2.3 eV in FWHM). This implies that the contribution for the natural angular width of the barium *K*-edge, 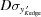, is about 382 µm (15.0 eV in FWHM).

The 386.6 µm contribution is then subtracted in quadrature from each data point using equation (4) and the result 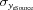 was plotted against XSR values [Fig. 6(*a*) ▸].

The divergence of the electron source, 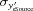, shown in Fig. 6(*b*) ▸, is obtained from equation (2) using a calculated value of 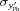 = 48.1 µrad and the experimentally determined 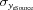 values.

It should be noted that the ps-BPM system not only measures the size and divergence but also the position and angle of the electron source at a single location with a single set of measurements. The results of all four values are summarized in Table 1 ▸ where 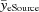 and 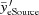 are the position and angle of the electron source, respectively.

### 3.4. Simulation to study ps-BPM sensitivity   

To assess the sensitivity of the system for measuring beam properties that determine the emittance as well as assessing other optical arrangements, ray-tracing simulations were performed.

The BM source was simulated with the built-in widget in *ShadowOui*. The nominal electron beam size input is 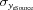 = 51.3 µm with divergence of 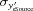 = 9.83 µrad. An energy bandwidth of 100 eV around the barium *K*-edge (37.441 keV) was used in the simulation to ensure coverage of the full phase and energy space of the system. To minimize statistical error, a total of 5 × 10^7^ rays were used in each calculation. The simulation error is defined as the RMS deviation of ten calculations.

The monochromator was simulated with the DCM widget in *ShadowOui* with the dispersion effect of the plane crystals taken into account. The *K*-edge filter absorption was calculated with a Python script that appropriately attenuates the rays after the monochromator. The transmitted rays through the filter were collected into a histogram at the detector plane, where the bin size represents the detector pixel size.

The simulated beam profiles (the histogram) with and without the *K*-edge absorption filter were then analyzed using the same data analysis process as the experimental results. The output of each calculation gives the unfiltered beam and *K*-edge filtered beam widths.

Calculations were performed with zero electron beam source size and divergence (zero emittance) to determine the photon, monochromator and *K*-edge contributions that are needed in equations (2) and (4).

Results obtained from ten bending magnet and ten zero emittance source simulations show expected measured values of 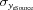 = 55.9 ± 1.6 µm (input value = 51.3 µm) and 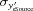 = 9.59 ± 0.35 µrad (input value = 9.83 µrad).

**Figure 7:**
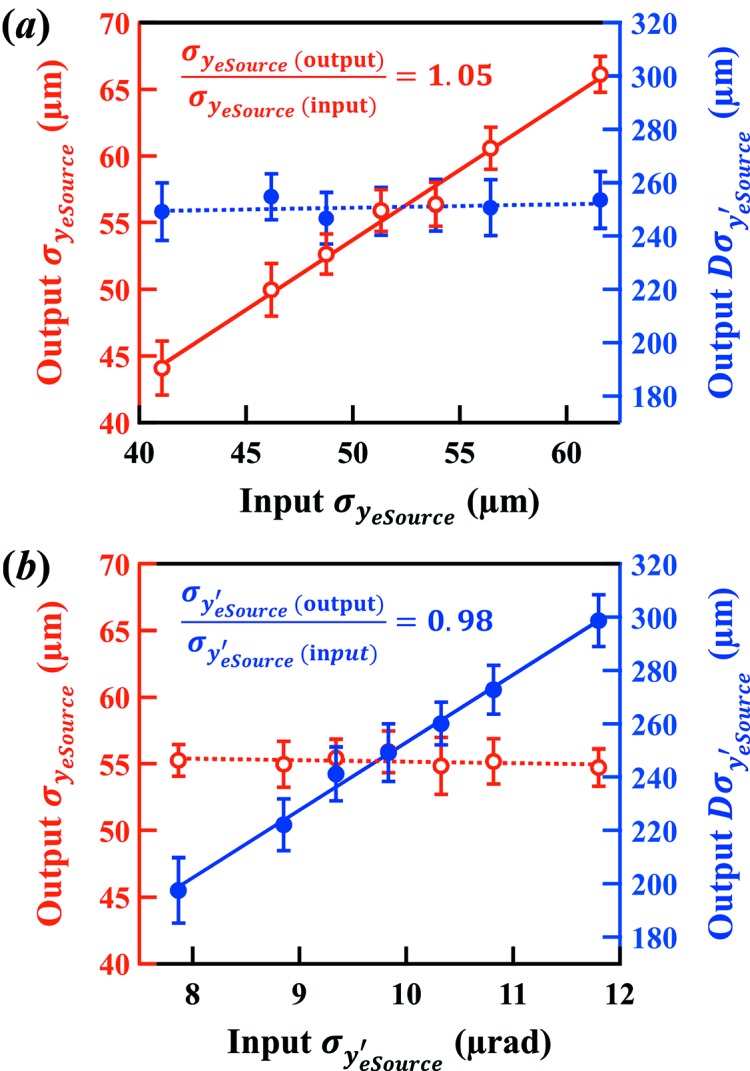
Simulation studies of the sensitivity of the ps-BPM system. Extracted electron source size (open markers) and divergence (closed markers) from different input values of (*a*) the electron source size and (*b*) the divergence.

For sensitivity studies, simulations were performed by varying the electron beam source size and divergence. The inputs 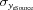 and 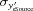 were scanned about their nominal values. The results were processed in the same way as described above and are plotted in Fig. 7 ▸.

The results indicate that the system can easily detect 5% changes in the source size and angle.

## 4. Discussion and conclusions   

The determination of the emittance properties from electron storage rings is becoming more important as sources are becoming brighter. This paper describes a new method of measuring the source size and divergence of a storage ring. The emittance of the source can then be determined if the beta function of the machine is known (Floettmann, 2003 ▸).

The ps-BPM system is capable of measuring the vertical electron beam source position, angular position, the vertical beam size and divergence at the same time. A model of the ps-BPM system has been developed for understanding how these parameters are extracted from measured beam and *K*-edge filtered widths. This understanding has been validated with commonly used modelling programs.

Specifically, the purpose of the simulation study was to (*a*) validate the data analysis, (*b*) validate the physical understanding of the system, (*c*) study the sensitivity of the system and (*d*) determine the contribution of the monochromator to the *K*-edge filtered width measurements.

Comparing the measurements of the pinhole based beamline with the ps-BPM system clearly shows that changing the beam size using the skew quads can be detected by both systems. The fact that the measured values are not the same is a consequence of the electron sources being located at different beta-functions.

It should be noted that this system measures the electron source properties in one direction (vertical) and requires a BM source.

The ps-BPM system measures the electron beam position and angle in real time (Samadi *et al.*, 2015 ▸). The measurement of the source size and divergence does require longer integration time (a few seconds in the current setup). The speed can be improved by using a wider horizontal BM fan and an optimized geometry (*e.g.* shorter source-to-detector distance).

Further studies are planned to calibrate the system by measuring the source size with different methods at the same beamline as the ps-BPM system.

## Figures and Tables

**Table 1:** Measured electron source size (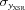) by a pinhole at the XSR beamline made as the vertical source size was changed using skew quadruples. Included are measurements by the ps-BPM system at the BMIT beamline of the electron source size (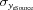), average source position (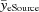), divergence (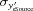), average angular position (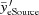), and the average beam position (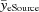 + 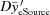)

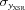 (µm)	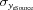 (µm)	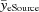 (µm)	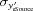 (µrad)	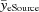 (µrad)	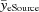 + 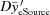 (µm)
83.6	98.0 ± 2.6	271 ± 8	21.11 ± 0.11	0.90 ± 0.61	294 ± 15
76.6	89.1 ± 2.8	277 ± 8	20.24 ± 0.08	0.53 ± 0.62	290 ± 16
70.7	84.2 ± 2.6	277 ± 8	19.41 ± 0.10	0.66 ± 0.63	294 ± 16
66.5	74.3 ± 3.1	283 ± 8	18.99 ± 0.08	0.33 ± 0.71	291 ± 18
65.2	75.3 ± 3.7	278 ± 8	18.80 ± 0.06	0.70 ± 0.65	297 ± 16
65.6	76.5 ± 2.1	276 ± 8	19.05 ± 0.10	0.73 ± 0.68	295 ± 17
82.4	96.1 ± 2.2	269 ± 8	21.02 ± 0.11	0.62 ± 0.62	285 ± 16
89.8	103.8 ± 3.1	266 ± 8	22.20 ± 0.15	0.80 ± 0.65	286 ± 16
98.2	111.8 ± 2.2	263 ± 8	23.56 ± 0.15	0.89 ± 0.62	286 ± 15
106.8	122.4 ± 1.4	261 ± 8	25.14 ± 0.12	0.76 ± 0.62	281 ± 15

## References

[bb1] Andersson, Å., Böge, M., Lüdeke, A., Schlott, V. & Streun, A. (2008). *Nucl. Instrum. Methods Phys. Res. A*, **591**, 437–446.

[bb2] Babanov, Y. A., Ryazhkin, A. V., Sidorenko, A. F. & Blaginina, L. A. (1998). *J. Struct. Chem.* **39**, 833–838.

[bb3] Bergstrom, J. C. & Vogt, J. M. (2008). *Nucl. Instrum. Methods Phys. Res. A*, **587**, 441–457.

[bb4] Borland, M., Abliz, M., Arnold, N., Berenc, T., Blednykh, A., Byrd, J., Calvey, J., Carter, J., Carwardine, J. & Cease, H. (2018). *9th International Particle Accelerator Conference (IPAC’18)*, 29 April–4 May 2018, Vancouver, BC, Canada, pp. 2872–2877.

[bb5] Buon, J. (1992). *CAS – CERN Accelerator School: 5th General Accelerator Physics Course*, 7–18 September 1992, Jyväskylä, Finland, pp. 89–116.

[bb6] Corbett, J., Huang, X., Li, C., Mitsuhashi, T., Wu, J., Xu, Y. & Zhang, W. (2017). *5th International Beam Instrumentation Conference (IBIC’16)*, 13–18 September 2016, Barcelona, Spain, pp. 237–240.

[bb7] DuMond, J. W. M. (1937). *Phys. Rev.* **52**, 872–883.

[bb8] Einfeld, D., Plesko, M. & Schaper, J. (2014). *J. Synchrotron Rad.* **21**, 856–861. 10.1107/S160057751401193X PMC4181637 25177977

[bb9] Elleaume, P., Fortgang, C., Penel, C. & Tarazona, E. (1995). *J. Synchrotron Rad.* **2**, 209–214. 10.1107/S0909049595008685 16714818

[bb10] Eriksson, M., van der Veen, J. F. & Quitmann, C. (2014). *J. Synchrotron Rad.* **21**, 837–842. 10.1107/S1600577514019286 25177975

[bb11] Floettmann, K. (2003). *Phys. Rev. ST Accel. Beams*, **6**, 034202.

[bb12] Golovchenko, J. A., Levesque, R. A. & Cowan, P. L. (1981). *Rev. Sci. Instrum.* **52**, 509–516.

[bb13] Guigay, J.-P., Zabler, S., Cloetens, P., David, C., Mokso, R. & Schlenker, M. (2004). *J. Synchrotron Rad.* **11**, 476–482. 10.1107/S0909049504024811 15496735

[bb14] Iida, K., Nakamura, N., Sakai, H., Shinoe, K., Takaki, H., Fujisawa, M., Hayano, H., Nomura, M., Kamiya, Y., Koseki, T., Amemiya, Y., Aoki, N. & Nakayama, K. (2003). *Nucl. Instrum. Methods Phys. Res. A*, **506**, 41–49.

[bb15] Kohn, V., Snigireva, I. & Snigirev, A. (2000). *Phys. Rev. Lett.* **85**, 2745–2748. 10.1103/PhysRevLett.85.2745 10991223

[bb16] Lyubomirskiy, M., Snigireva, I., Kohn, V., Kuznetsov, S., Yunkin, V., Vaughan, G. & Snigirev, A. (2016). *J. Synchrotron Rad.* **23**, 1104–1109. 10.1107/S160057751601153X 27577763

[bb17] Mitsuhashi, T. (1999). *Beam Measurement: Proceedings of the Joint US-CERN-Japan-Russia School on Particle Accelerators*, 11–20 May 1998, Montreux, and CERN, Switzerland, pp. 399–427. Singapore: World Scientific.

[bb18] Naito, T. & Mitsuhashi, T. (2006). *Phys. Rev. ST Accel. Beams*, **9**, 122802.

[bb19] Peatman, W. B. & Holldack, K. (1998). *J. Synchrotron Rad.* **5**, 639–641. 10.1107/S0909049597017068 15263604

[bb21] Rebuffi, L. & Sánchez del Río, M. (2016). *J. Synchrotron Rad.* **23**, 1357–1367. 10.1107/S1600577516013837 PMC5298219 27787241

[bb20] Rebuffi, L. & Sánchez del Río, M. (2017). *Proc. SPIE*, **10388**, 103880S.

[bb22] Renner, T., Padmore, H. & Keller, R. (1996). *Rev. Sci. Instrum.* **67**, 3368.

[bb23] Samadi, N., Bassey, B., Martinson, M., Belev, G., Dallin, L., de Jong, M. & Chapman, D. (2015). *J. Synchrotron Rad.* **22**, 946–955. 10.1107/S1600577515007390 PMC4489536 26134798

[bb24] Schwinger, J. (1949). *Phys. Rev.* **75**, 1912–1925.

[bb25] Shi, X., Marathe, S., Wojcik, M. J., Kujala, N. G., Macrander, A. T. & Assoufid, L. (2014). *Appl. Phys. Lett.* **105**, 041116.

[bb26] Takano, S., Masaki, M. & Ohkuma, H. (2006). *Nucl. Instrum. Methods Phys. Res. A*, **556**, 357–370.

[bb27] Tavares, P. F., Leemann, S. C., Sjöström, M. & Andersson, Å. (2014). *J. Synchrotron Rad.* **21**, 862–877. 10.1107/S1600577514011503 PMC4181638 25177978

[bb28] Thomas, C., Rehm, G., Martin, I. & Bartolini, R. (2010). *Phys. Rev. ST Accel. Beams*, **13**, 022805.

[bb29] Warren, B. E. (1990). *X-ray Diffraction.* Courier Corporation.

[bb30] Weitkamp, T., Chubar, O., Drakopoulos, M., Snigireva, I., Snigirev, A., Schroer, C., Guenzler, F. & Lengeler, B. (2000). *Proceedings of the Seventh European Particle Accelerator Conference (EPAC2000)*, 26–30 June 2000, Austria, Vienna, pp. 1824–1826.

[bb31] Wysokinski, T. W., Chapman, D., Adams, G., Renier, M., Suortti, P. & Thomlinson, W. (2007). *Nucl. Instrum. Methods Phys. Res. A*, **582**, 73–76.

[bb32] Wysokinski, T. W., Chapman, D., Adams, G., Renier, M., Suortti, P. & Thomlinson, W. (2013). *J. Phys. Conf. Ser.* **425**, 072013.

[bb33] Zachariasen, W. H. (1945). *Theory of X-ray Diffraction in Crystals.* New York: John Wiley.

